# Magnifying narrow-band imaging of gastric mucosal morphology predicts the H. pylori-related epigenetic field defect

**DOI:** 10.1038/s41598-017-03294-8

**Published:** 2017-06-08

**Authors:** Tomomitsu Tahara, Jumpei Yamazaki, Sayumi Tahara, Masaaki Okubo, Tomohiko Kawamura, Noriyuki Horiguchi, Takamitsu Ishizuka, Mitsuo Nagasaka, Yoshihito Nakagawa, Tomoyuki Shibata, Makoto Kuroda, Naoki Ohmiya

**Affiliations:** 1 Department of Gastroenterology, Fujita Health University School of Medicine, Toyoake, Japan; 2 Laboratory of Molecular Medicine, Hokkaido University Graduate School of Veterinary Medicine, Sapporo, Japan; 3 Department of Diagnostic Pathology I, School of Medicine, Fujita Health University, Toyoake, Japan

## Abstract

DNA methylation is associated with “field defect” in the gastric mucosa. To characterize “field defect” morphologically, we examined DNA methylation of non-neoplastic gastric mucosa in relation to their morphology seen by narrow-band imaging (NBI) with magnifying endoscopy. Magnifying NBI of non-neoplastic gastric body was classified as follows: normal—small and round pits with uniform subepithelial capillary networks; type 1—a little enlarged round pits with indistinct subepithelial capillary networks; type 2—remarkably enlarged pits with irregular vessels; and type 3—clearly demarcated oval or tubulovillous pits with bulky coiled or wavy vessels. Methylation of nine candidate genes (*MYOD1*, *SLC16A12*, *GDNF*, *IGF2*, *MIR 124A1*, *CDH1*, *PRDM5*, *RORA* and *MLF1*) were determined by bisulfite pyrosequencing. Infinium HumanMethylation450 array was used to characterize the methylation of >450,000 CpG sites. Mean Z score methylation of nine genes positively correlated with the changes of mucosal patterns from normal to types 1, 2, and 3 (*P* < 0.0001). Genome-wide analysis showed that development of mucosal patterns correlated with methylation accumulation especially at CpG islands. Genes with promoter CpG islands that were gradually methylated with the development of mucosal patterns significantly enriched the genes involved in zinc-related pathways. The results indicates that gastric mucosal morphology predicts a “field defect” in this tissue type. Accumulation of DNA methylation is associated with “field defect” in the non-neoplastic gastric mucosa. Endoscopic identification of “field defect” has important implications for preventing gastric cancer. Our results suggest that magnifying NBI of gastric mucosal morphology predicts a “field defect” in the gastric mucosa.

## Introduction

Promoter CpG island (PCGI) methylation and subsequent transcriptional repression are important mechanisms in many types of cancers^1^, while this phenomenon is also observed in *H. pylori*-infected non-neoplastic gastric mucosa^1–3^. This phenomenon can be explained by the concept of an “epigenetic-field-defect”, which is linked to gastric cancer predisposition. Several factors are shown to be associated with this molecular change^1^, while gastric mucosa morphologic features of “epigenetic-field-defect” is unclear.

The narrow-band imaging (NBI) uses narrow bands of red, blue, and green filters to obtain a clear visualization of mucosal and capillary patterns^4^. Combining magnifying endoscopy and the NBI system can lead to visualizing microscopic mucosal structures and its capillary patterns more clearly, which can better predict the histopathological state of the endoscopic lesion throughout the gastrointestinal tract^5–8^.

We have reported that gastric mucosal patterns visualized with magnifying NBI correlate well with the histological degree of *Helicobacter pylori* (*H. pylori*)-related gastritis^9^. Consequent results also demonstrated that the NBI patterns in uninvolved gastric mucosa are linked to their gastric cancer risk^10^. *H. pylori* infection triggers various genotoxic and epigenetic changes in the gastric mucosa, which constitute the earliest steps toward gastric cancer predisposition^2, 3, 11^. Gastric mucosal patterns seen with magnifying NBI may therefore provide information to estimate the “field defect” in the stomach.

Since endoscopic identification of “field defect” has important implications for preventing gastric cancer, we performed comprehensive DNA methylation characterization of non-neoplastic gastric mucosa in relation to their magnifying NBI features.

## Results

### Clinicopathological characteristics

The clinicopathological characteristics of the subjects are shown in Table 1. The prevalence of *H. pylori* infection, gastric cancer, inflammatory mucosa and atrophic mucosa in different magnifying NBI patterns are also shown in Supplementary Table 2. None of the samples with a normal pattern did not have *H. pylori* infection, gastric cancer, inflammatory mucosa or atrophic mucosa. *H. pylori* infection was frequent in types 1 and 2, while the prevalence of inflammatory mucosa was more frequent in type 2 than in type 1. The type 3 pattern was characterized as atrophic mucosa and was frequently observed in gastric cancer patients. All of these observations were in line with our previous report^9, 10^.

**Table 1:** Clinicopathological characteristics of subjects.

Variables	
Total number	94
Age: median (range)	64.5 (22–87)
Gender: male/female	66/28
*H. pylori* positives	52/93*
Ulcer disease	11
GU/DU	4/7
Gastric cancer	23

*For one case, *H. pylori* status was not determined. GU, gastric ulcer; DU, duodenal ulcer.

### Candidate promoter methylation analysis in relation to magnifying NBI patterns

The methylation status of nine genes was determined by bisulfite pyrosequencing.

**Figure 1:**
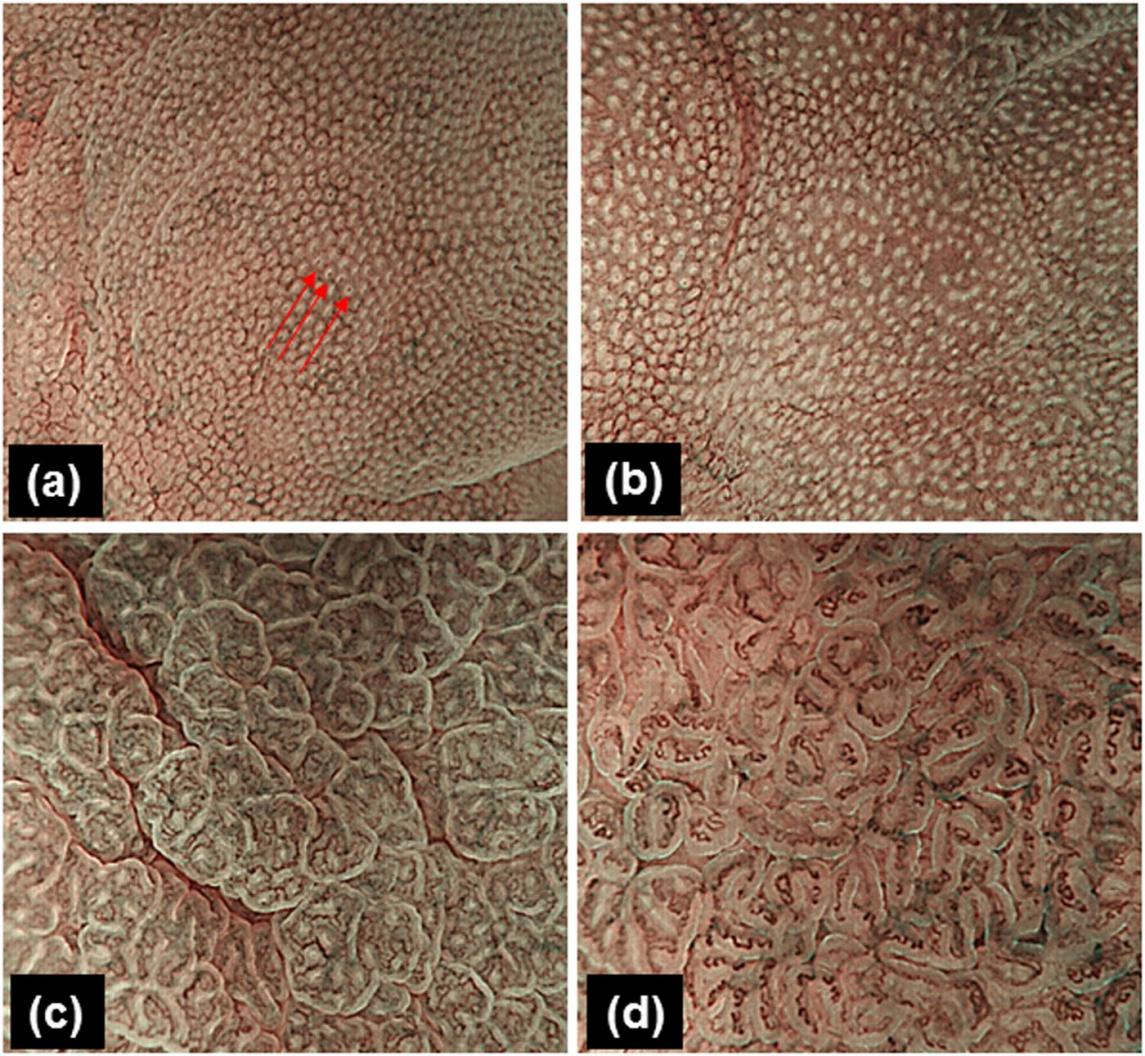
Typical cases of 1 normal and 3 gastric mucosal patterns in the corpus seen with magnifying NBI endoscopy (Tahara *et al*. Gastrointest Endosc. 2009;70:246-53). Normal: small, round pits surrounded by subepithelial capillary networks (red arrows). Type 1: slightly enlarged, round pit with unclear or irregular subepithelial capillary networks. Type 2: obviously enlarged, oval or prolonged pit with increased density of irregular vessels. Type 3: well-demarcated, oval or tubulovillous pit with clearly visible coiled or wavy vessels. subepithelial capillary networks.

**Figure 2:**
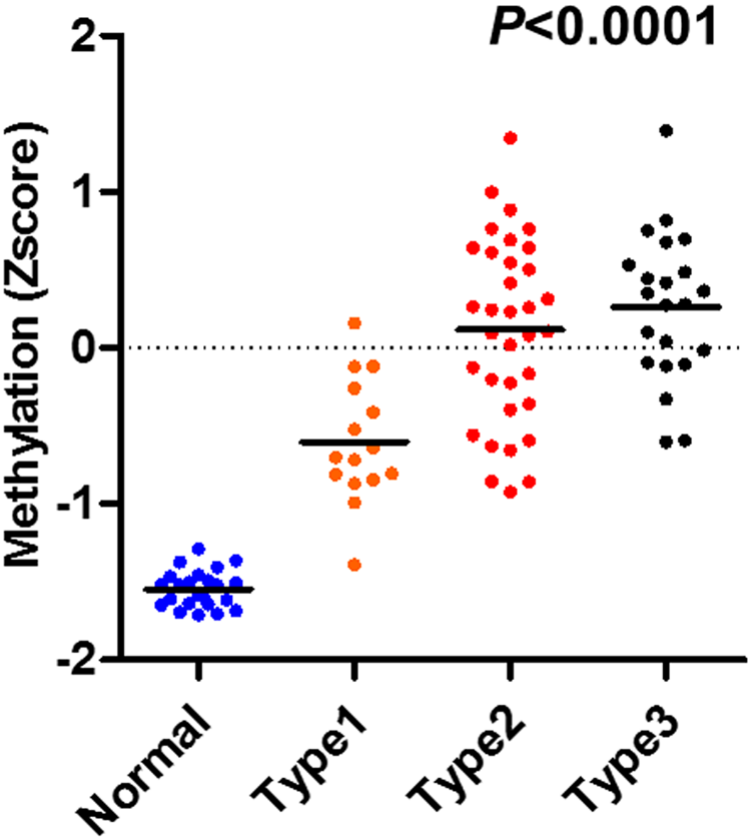
Mean Z score methylation of nine candidate genes in relation to the NBI mucosal patterns. Statistical analysis was performed using the Spearman correlation analysis.

**Figure 3:**
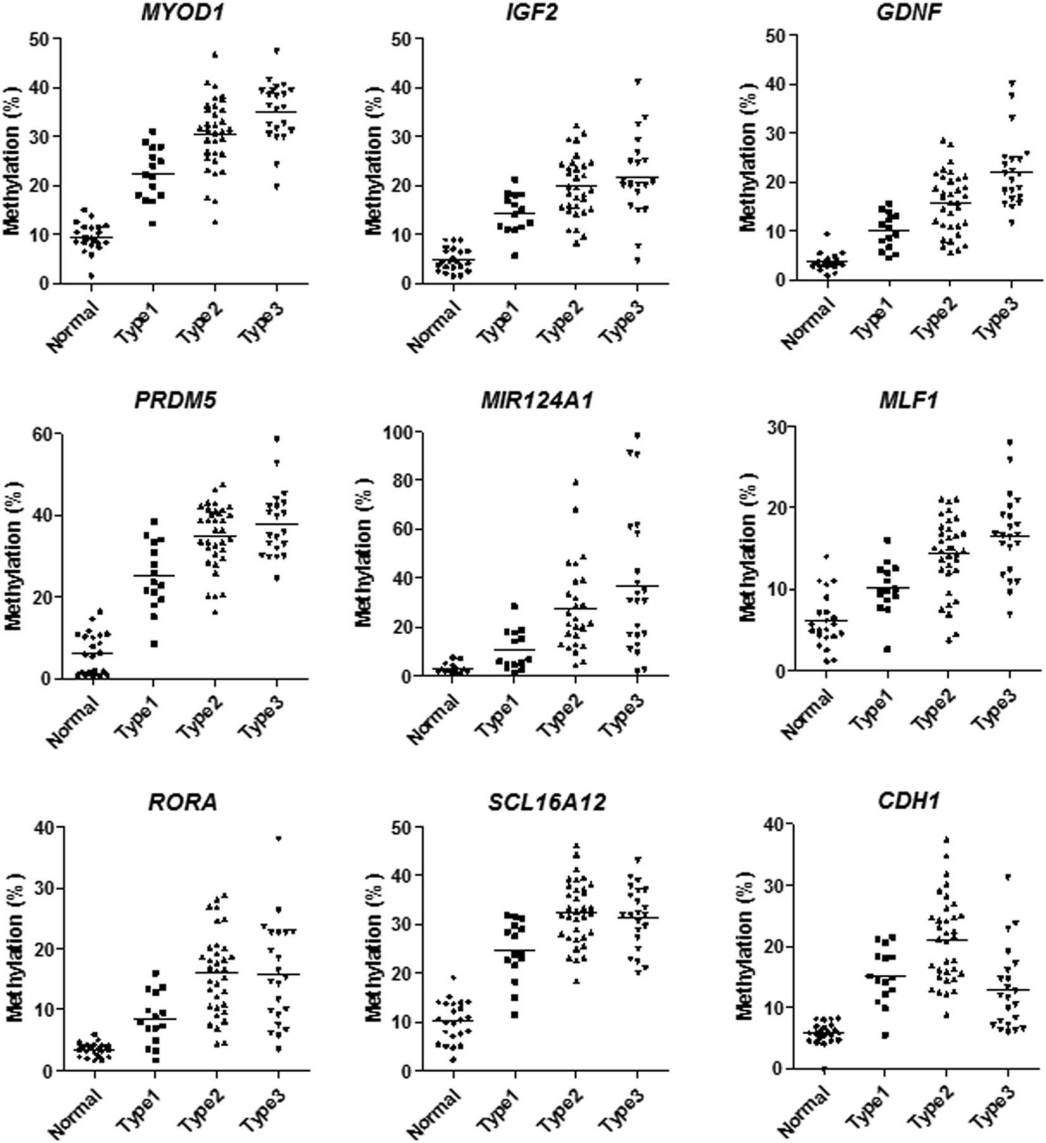
Methylation status of individual genes in relation to the NBI mucosal patterns (all *P* values < 0.0001). Statistical analysis was performed using one-way ANOVA.

These genes were reported to be methylated in gastric cancer (*RORA*, *GDNF*, *PRDM5* and *MLF1*, ref. 12) or in *H. pylori*-infected gastric mucosa (*MYOD1*, *SLC16A12*, *IGF2*, *MIR124A1* and *CDH1*, ref. 13). Initially, we averaged the methylation levels of these nine genes as the mean Z score in relation to the magnifying NBI patterns. The representative NBI patterns of the normal to types 1, 2, and 3 are shown in Fig. 1. The result demonstrated that the change of mucosal patterns from normal to types 1, 2, and 3 was closely associated with higher mean Z score methylation (*P* < 0.0001, Fig. 2). When we focused on individual genes, the methylation levels of six genes (*MYOD1*, *GDNF*, *IGF1*, *MLF1*, *MIR124A1*, and *PRDM5*) increased with the change of mucosal patterns from normal to types 1, 2, and 3, while the methylation levels of two genes increased from normal to types 1 and 2, and the methylation levels of these two genes were the same in type 3 (*SLC16A12* and *RORA*). In contrast, the methylation of *CDH1* became higher between normal to types 1 and 2, while the methylation of *CDH1* went down in type 3 (Fig. 3). We next performed univariate and multivariate analyses to assess the factors related to DNA methylation in the gastric mucosa. NBI pattern, age, gender, gastric cancer occurrence, *H. pylori* infection, inflammatory mucosa or atrophic mucosa were included for this analysis. Because the mean Z score methylation of all nine genes in the gastric mucosa presented an approximately Gaussian distribution, with over-representation of methylation-high cases (data not shown), we set cut-off values of 0.15 (mean Z score methylation) for the definition of methylation-high cases. The univariate analysis revealed that NBI pattern (odds ratio: 4.04, 95% confidence interval: 2.12-7.7, *P* < 0.0001), male gender (odds ratio: 4.15, 95% confidence interval: 1.29-13.34, *P* = 0.02), gastric cancer occurrence (odds ratio: 3.83, 95% confidence interval: 1.43-10.23, *P* = 0.007), inflammatory mucosa (odds ratio: 7.77, 95% confidence interval: 2.77-21.77, *P* < 0.0001) and atrophic mucosa (odds ratio: 4.97, 95% confidence interval: 1.77-13.90, *P* = 0.002) were significantly associated with methylation-high cases (Supplementary Table 3). The multivariate analysis of these factors revealed that only the NBI pattern was an independent factor for DNA methylation in the gastric mucosa (odds ratio: 8.55, 95% confidence interval: 2.00-36.6, *P* = 0.004, Table 2).

**Table 2:** Multivariate analysis assessing the factors related to methylation-high cases.

Variables	Odds ratio (95% confident interval)	*P* value
NBI pattern	8.55 (2.00–36.6)	0.004
Gender (male)	3.76 (0.89–15.97)	0.07
Gastric cancer occurrence	0.30 (0.05–1.92)	0.2
Inflammatory mucosa	1.93 (0.55–6.74)	0.3
Atrophic mucosa	0.39 (0.05–3.14)	0.38

*Methylation high, mean Z score of methylation >0.15. *Mean Z score of methylation more than 0.15 was considered methylation high.

### Genome-wide methylation analysis

**Figure 4:**
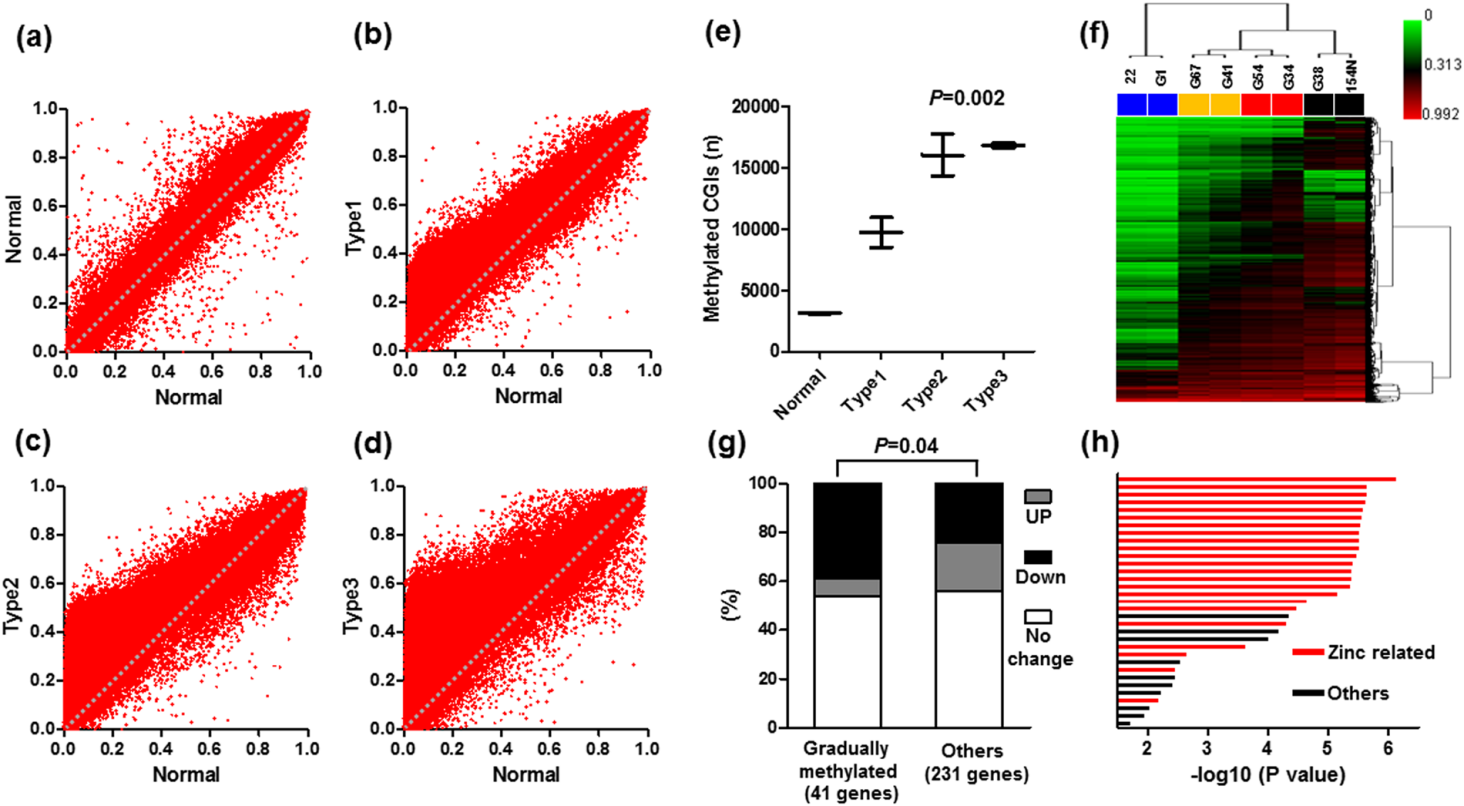
Genome-wide methylation analysis in CpG islands (CGIs). (**a**–**d**) The development of mucosal patterns from normal to types 1, 2, and 3, and methylation accumulation in CGIs. (**e**) Mean number of methylated CGIs from normal to types 1, 2, and 3. Statistical analysis was performed using one-way ANOVA. (**f**) Unsupervised hierarchical clustering analysis of 10% of the most variant probes. Blue, yellow, red and black boxes represent normal and type 1, 2, and 3 samples, respectively. Sample ID numbers are listed above the boxes. (**g**) Gene expression fold change between normal to type 3 among genes codified by the gradually methylated PCGIs with the development of mucosal patterns from normal to types 1, 2, and 3 (41 genes) and others (231 genes). UP, upregulated > two fold; Down, downregulated > two fold; Statistical analysis was performed using chi square test. (**h**) Gene ontology analysis of genes codified by PCGIs that were gradually methylated with the development of mucosal patterns from normal to types 1, 2, and 3. The red bars represent significant enrichment of Zinc-related pathways. The black bar represents significant enrichment for other pathways.

To comprehensively observe the methylation changes in relation to the NBI patterns, the Infinium HumanMethylation450 BeadChip array was used. We examined eight samples (n = 2, for each NBI pattern). Based on the GRCh37/hg19, we first checked the methylation status, dividing sites into CpG islands (CGI: n = 145842) and non-CpG islands (NCGI: n = 328022). We found that accelerated methylation at CGI was shown in relation to the change of mucosal patterns from normal to types 1, 2, and 3 (Fig. 4a–d). When a gain of methylation was defined as a methylation level ≥30% (β-value ≥ 0.3), the mean number of methylated sites significantly increased in relation to the change of mucosal patterns from normal to types 1, 2, and 3 (3126, 9730, 16060 and 16833, respectively, *P* = 0.002, Fig. 4e). Additionally, unsupervised clustering analysis using 10% of the most variant probes very clearly distinguished normal and type 1, 2, and 3 patterns (Fig. 4f). Although unsupervised clustering analysis of NCGI also distinguished normal and type 1, 2, and 3 patterns (Fig. S1f), which was not striking compared to the CGI (Fig. S1a–d) because more than half of the CpG sites were methylated in all NBI patterns in the NCGI. The number of methylated sites was not significantly different among different NBI patterns (Fig. S1e). The clustering analysis of CGI and NCGI, dividing sites into promoter (PCGI: n = 1653 and NPCGI: n = 20222) and outside promoter (PNCGI: n = 2573 and NPNCGI: n = 34717) all distinguished normal and type 1, 2, and 3 patterns (Fig. S2a–d). In detail, the change of mucosal patterns from normal to types 1, 2, and 3 was associated with hypermethylation in PCGI, NPCGI and NPNCGI (Fig. S2a,b,d), while the analysis of PNCGI showed that the methylation was increased from types 2, 1 and normal and type 3 (Fig. S2c). Next, we further focused the analysis on PCGI methylation because of its influence on gene expression. Through the clustering analysis of PCGIs, we identified 210 PCGIs for which the methylation accumulation is closely associated with the change of mucosal patterns from normal to types 1, 2, and 3. From these 210 sites, we chose 112 sites that represented single genes (56 genes), and we investigated the association with the gene expression status from the microarray data. Among the genes for which the expression data was available (41 out of 56 genes), 16 (39%) and 3 genes (7.3%) were downregulated and upregulated more than two-fold, respectively, and the other 22 genes (53.7%) did not show any fold change between normal and type 3. In contrast, the same analysis of the remaining PCGIs (272 genes) showed that 55 (23.8%) and 47 genes (20.3%) were downregulated and upregulated more than two-fold, respectively, and the other 129 genes (55.8%) did not show any fold change between normal and type 3. The genes that were gradually methylated with the change of mucosal patterns from normal to types 1, 2, and 3 were more closely associated with downregulation compared with the others (Fig. 4g, *P* = 0.04). The clustering analysis of PCGI also identified the groups of genes that were strikingly hypermethylated (63 genes) and hypomethylated (86 genes) only in type 3. However, these groups were not associated with gene expression (Fig. S3, *P* = 0.78). We next asked whether there was any functional enrichment for genes for which the methylation was associated with the development of mucosal patterns. Gene ontology analysis using DAVID revealed striking enrichment of Zinc-related pathways in the gradually methylated genes with the development of mucosal patterns. A total of 23 out of 33 significant enrichments were Zinc-related pathways and most of them corresponded to C2H2-type (classical) zinc fingers (Fig. 4h and Table S4). In contrast, such associations were not found for groups of genes that were strikingly hypermethylated and hypomethylated only in type 3 (Tables S5, S6).

## Discussion

Initially, we investigated PCGI methylation of nine gastric cancer or *H. pylori-*related genes among different NBI mucosal patterns. Change of mucosal patterns from normal to types 1, 2, and 3 correlated with higher mean Z score methylation levels of these nine genes. Previous studies demonstrated that PCGI methylation occurs in non-neoplastic gastric mucosa with the severity of *H. pylori*-related gastritis 1. Indeed, the univariate analysis identified that inflammatory mucosa and atrophic mucosa were also significantly associated with PCGI hypermethylation. However, the multivariate analysis revealed that the NBI pattern, but not the histological inflammation or atrophy was associated with DNA methylation status as an independent factor. The NBI patterns reflect the pathological conditions of *H. pylori-*related gastritis, considering both the inflammatory and atrophic states. The change of NBI mucosal patterns from normal to types 1 and 2 simply correlates with the degree of inflammation, while the type 3 pattern is associated with atrophy and metaplasia^9^. Therefore, the NBI mucosal patterns would correlate more strongly with methylation accumulation than with histological inflammation or atrophy alone. Many studies have suggested that DNA methylation in the non-neoplastic gastric mucosa is a risk marker for gastric cancer^1, 14, 15^. We reported that the NBI mucosal features well correlated with gastric cancer occurrence and lower pepsinogen I/II ratios^9, 10^, which is an indicator for the spread of gastric atrophy^16^. On the other hand, our current result demonstrated that NBI mucosal patterns was the factor most associated with hypermethylation rather than gastric cancer occurrence. This indicates that the methylation change is closely linked to the NBI patterns within the focal points in the stomach. But it is also possible that assessing the spread of NBI patterns in the stomach may be informative for gastric cancer risk estimation. Therefore, our result needs to be evaluated by the prospective study using larger cohort to see whether NBI endoscopy is useful for gastric cancer risk prediction.

Our result showed that methylation status of individual genes suggested that methylation changes in relation to the NBI mucosal patterns appeared to be of three different types. One is a linear increase with the change of mucosal patterns from normal to types 1, 2, and 3 (*MYOD1*, *GDNF*, *IGF1*, *MLF1*, *MIR124A1*, and *PRDM5*). The second is an increase from normal to types 1 and 2, while maintained at the same level in type 3 (*SLC16A12* and *RORA*). The third is an increase with the change of mucosal patterns from normal to types 1 and 2, but a decrease in type 3 (*CDH1*). The majority of methylation changes appeared to occur in parallel with the development of mucosal patterns, reflecting the consequence of chronic inflammation on the development of gastric atrophy. In contrast, the methylation status of *CDH1* seems to be strongly associated with the inflammatory status rather than atrophy. In this study, the majority of patients of type 3 were *H. pylori-*negative (17/22, 76.2%). Because development of atrophic gastritis leads to the disappearance of *H. pylori* ^17^, which would show rather mild inflammation, this may be the reason for the lower methylation of *CDH1* in type 3 than in type 2. However, the methylation status of *CDH1* remained higher compared to normal. This remaining methylation would reflect past exposure to *H. pylori*, which should be considered as a potential risk for gastric cancer.

To observe comprehensive methylation changes through the change of NBI mucosal patterns, we performed genome-wide methylation analysis. Our result showed that a high rate of hypermethylation occurred in parallel with the development of NBI mucosal patterns from normal to types 1, 2, and 3, especially at CGI. Although clustering of 10% of the most variant probes at NCGI also discriminated normal and type 1, 2, and 3 patterns, the number of methylated sites was not significantly different among those NBI patterns because more than half of the CpG sites were methylated in all NBI patterns in the NCGI, which would be a common phenomenon in many tissues^18^. Our result suggest that in the gastric mucosa, alterations in methylation related to epigenetic field defects are characterized as hypermethylation in CGI, which is also supported by the methylation analysis of nine candidate gene by the pyrosequencing. The genome wide analysis have identified groups of PCGIs, for which the methylation accumulation is closely associated with the change of mucosal patterns from normal to types 1, 2, and 3. The microarray data demonstrated that this methylation is significantly associated with transcriptional repression. Although other groups of PCGIs such as strikingly hypermethylated and hypomethylated PCGIs were only identified in type 3, these groups were not associated with gene expression status. This finding emphasizes the biological importance of genes that were gradually methylated with the change of mucosal patterns from normal to types 1, 2, and 3 in relation to methylation-related downregulation. We have also shown that gradual methylation during the development of mucosal patterns frequently codified genes involved in Zinc-related pathways. Importantly, hypermethylation of genes involved in Zinc-related pathways have been reported in several cancers including gastric cancers^19–21^. The result emphasizes the importance of a better understanding of pathway-specific molecular changes in the development of NBI mucosal patterns, which reflect *H. pylori*-related tumorigenesis. The magnifying endoscopic feature of colorectal cancer has been reported to be associated with its genetic and epigenetic status, reflecting its prognosis and response to treatment. Our current result also demonstrated the reliability of the gastric mucosal morphology visualized by magnifying NBI to estimate the “field defect” from a molecular view point. We believe that our findings provide salient information for many endoscopist to further advance clinical trials evaluating the reliability of magnifying NBI endoscopy in this field.

## Materials and Methods

### Study participants

We included study participants were prospectively, in the Endoscopy Center of Fujita Health University from January, 2013 to December, 2014. We invited one hundred twenty-six participants, and all of them agreed to participate in the study. These participants included 30 patients with gastric cancer who visited to Fujita Health University hospital for laparoscopic surgery or for endoscopic mucosal dissection. Other participants underwent upper gastroscopy for various reasons, such as annual screening for malignancy, secondary check-up after barium X-ray examination for suspicious of gastric diseases, and abdominal symptoms. Patients with severe systemic disease, advanced chronic liver disease; those who were taking non-steroidal anti-inflammatory drugs, proton-pump inhibitors, or H2 receptor antagonists; those who had history of *H. pylori* eradication therapy; and those with a history of gastrectomy. Fujita Health University School of Medicine approved all the study protocol, and written informed consent for study enrolment as well as publish identifying information/images was obtained from all participants. All study methods were performed in accordance with the relevant guidelines and regulations.

### Endoscopic procedure, classification of magnifying NBI patterns, detection of H. pylori infection

All participants underwent esophagogastroduodenoscopy (EGD) using a magnifying video endoscope (Olympus GIF-H260Z and a CV260SL/CV290SL endoscopic system, Olympus Medical Systems) and the non-neoplastic mucosa of the greater curvature in the gastric corpus were carefully observed by using the complete magnification coupled with a NBI light source. According to our previous study^9^, most predominant gastric mucosal patterns visualized by the magnifying NBI in the greater curvature of uninvolved gastric corpus were divided into the following categories: normal—small and round pits with uniform subepithelial capillary networks; type 1—a little enlarged round pits with indistinct subepithelial capillary networks; type 2—remarkably enlarged pits with irregular vessels; and type 3—clearly demarcated oval or tubulovillous pits with bulky coiled or wavy vessels (Fig. 1). This classification system has been reported to clearly distinguish histological degree of chronic gastritis and to correlate with gastric cancer occurrence^9, 10^. The classification of mucosal patterns among each case was based on the most predominant magnifying NBI pattern. The most predominant magnifying NBI pattern was taken as endoscopic pictures, and biopsy specimens were taken from targeted site. We obtained at least two biopsies from the targeted site. Using one biopsy specimen, the degrees of neutrophil and mononuclear cell infiltration, atrophy, and metaplasia was assessed according to the updated Sydney system^22^ and scored from 0 (normal) to 3 (marked). We defined a neutrophil infiltration score ≥2 or a mononuclear cell infiltration ≥2 as inflammatory mucosa. We also defined an atrophy score ≥2 or a metaplasia score ≥2 as atrophic mucosa. The other biopsy was immediately frozen and stored at −80 °C for the molecular experiment. Histology biopsy specimen, serum titer, and urea breath test were used for diagnosing *H. pylori* infection. A positive result of any of these tests was diagnosed as *H. pylori* infection, and a negative result of any of these tests was diagnosed as *H. pylori-*negative. After EGD procedure, an additional 32 participants were excluded due to inadequate image quality. Finally, 94 participants, including 23 patients with gastric cancer and 71 patients without evidence of cancers, were included in this study. All endoscopic observations and classification of NBI patterns were performed by 1 expert endoscopist (T. T.). The prevalence of NBI patterns in 94 participants was 23 (24.5%) normal, 15 (15.9%) type1, 34 (36.2%) type 2, and 22 (23.4%) type 3. The interobserver concordance using representative endoscopic pictures from all participants demonstrated good κ coefficient values greater than 0.75 for all patterns across an additional two expert endoscopists (M.O. and T.S.).

### Selection of candidate genes and PCGI methylation analysis by bisulfite pyrosequencing

Genomic DNA was extracted from the frozen gastric samples using the standard protein precipitation method. Bisulfite pyrosequencing was used to quantify PCGI methylation of nine genes. The selection of the nine genes were based on the frequency of methylation in gastric cancer (*RORA*, *GDNF*, *PRDM5* and *MLF1*, ref. 13) or *H. pylori*-infected gastric mucosa (*MYOD1*, *SLC16A12*, *IGF2*, *MIR124A1* and *CDH1*, Tahara *et al*. paper for submission). Bisulfite-treated genomic DNA was used to evaluate the methylation status by bisulfite pyrosequencing. Bisulfite treatment of DNA was carried out using an EZ DNA Methylation Kit (Zymo Research) according to the manufacturer’s protocol. Pyrosequencing was carried out using a PSQ24 system with Pyro-Gold reagent Kit (QIAGEN, Tokyo, Japan), and the results were analyzed using PyroMark Q24 software (QIAGEN). The primers used for pyrosequencing are listed in Supplementary Table 1.

### Genome-wide methylation analysis

We performed array-based DNA methylation analysis using the Infinium HumanMethylation450 BeadChip array, which allows us to query the methylation status of > 450,000 CpG sites within the genome and to cover 99% of RefSeq genes. Genomic DNA samples from eight patients (n = 2, for each NBI pattern) were used for this analysis. Bisulfite modification of genomic DNA was performed using an EZ DNA Methylation Kit (Zymo Research). The samples were then run on an Infinium HumanMethylation450K BeadChip (Illumina) and scanned on an Illumina iScan instrument according to the manufacturer’s instructions. The methylation values for individual CpG sites in each sample were obtained as β-values. The β-value generated for each CpG locus measure the intensity of methylated (β = 1) and unmethylated probes (β = 0). The β-value is a continuous variable that is calculated by dividing the intensity of the methylated beads by the combined intensity, and these values range from 0 to 1. Genomic regions were defined according to National Center for Biotechnology Information coordinates downloaded from the University of California Santa Cruz website in February, 2009 (GRCh37/hg19). We removed probes that were targeted for annotated SNP (dbSNP137) as well as for either the X or Y chromosomes. Information regarding CpG islands and promoters (surrounding a gene transcription start site) was also obtained based on the GRCh37/hg19.

### Clustering analysis

Unsupervised clustering analysis using ArrayTrack™ (http://www.fda.gov/ScienceResearch/BioinformaticsTools/Arraytrack/) was performed to identify distinct subgroups based on methylation status.

### Gene Ontology analysis

Functional enrichment of methylated genes was determined by gene ontology analysis using DAVID Bioinformatics Resources 6.7 (http://david.abcc.ncifcrf.gov/). P values were corrected for multiple hypothesis testing using the Benjamini method.

### Gene expression analysis

For two frozen samples (one normal and one type 3 pattern), total RNA was extracted using TRI reagent. cRNA was synthesized, amplified and labeled using the Low Input Quick Amp Labeling Kit (Agilent Technologies). Labeled cRNA was then purified using the RNeasy Mini Kit (Qiagen). Hybridization mixes were prepared using the Gene Expression Hybridization Kit (Agilent Technologies). A total of 600 ng Cy3-labeled sample cRNAs were hybridized on SurePrint G3 human GE 8 × 60 K microarrays (Agilent Technologies). The slide was washed according to the manufacturer’s specifications using the Gene Expression Wash Buffer Kit (Agilent Technologies). The slide was scanned using an Agilent G2539A scanner (Agilent Technologies) and pre-processed using the Agilent Feature Extraction Software, version 10.10.1.1 (Agilent Technologies). Pre-processed data were then analyzed using GeneSpring GX software, version 13.0.0 (Agilent Technologies). All arrays met the minimum Agilent QA/QC standards. All kits were used according to the manufacturer’s protocol.

### Statistical analysis

Continuous variables among more than two groups were assessed using one-way ANOVA. Categorical variables among different groups were assessed using chi square test. Univariate and multivariate analyses were also performed to assess the factors related to PCGI methylation. A P value < 0.05 was considered statistically significant.

## Electronic supplementary material

Dataset 1
