# Supplementary material for: Magnifying narrow-band imaging of gastric mucosal morphology predicts the H. pylori-related epigenetic field defect
Source: Sci Rep. 2017 Jun 8;7:3090. doi: 10.1038/s41598-017-03294-8 (PMC5465184; doi:10.1038/s41598-017-03294-8)
Supplement: Supplementary file 1 — Dataset 1 [file 41598_2017_3294_MOESM1_ESM.doc]

***Title: Magnifying narrow-band imaging of gastric mucosal morphology predicts the H. pylori-related epigenetic field defect***

Authors: Tomomitsu Tahara, PhD,*1,4, Jumpei Yamazaki, PhD, 2,4, Sayumi Tahara MD, 3, Masaaki Okubo, PhD, 1, Tomohiko Kawamura, MD, 1, Noriyuki Horiguchi, MD, 1, Takamitsu Ishizuka1, Mitsuo Nagasaka, PhD, 1, Yoshihito Nakagawa, PhD, 1, Tomoyuki Shibata, PhD, Makoto Kuroda PhD, 3, Naoki Ohmiya, PhD, 1

1 Department of Gastroenterology, Fujita Health University School of Medicine, Toyoake,

2 Laboratory of Molecular Medicine, Hokkaido University Graduate School of Veterinary Medicine

3 Department of Diagnostic Pathology I, School of Medicine, Fujita Health University, Toyoake, Japan

4 These authors contributed equally to this work

*Author for correspondence:

1–98 Dengakugakubo Kutsukake-cho, Toyoake, Aichi, 470–1192, Japan

Tel.: +81 562 93 9240

Fax: +81 562 93 8300

**Supplementary Tables and Figures**

**Supplementary Table 1. Primer sequences used for pyrosequencing**

| Assay name | Forward primer  (1st step PCR) sequence | Reverse primer (1st step PCR) sequence | Forward primer  (2nd step PCR) sequence | Reverse primer (2nd step PCR) sequence | Sequencing primer sequence |
| --- | --- | --- | --- | --- | --- |
| *RORA* | TTTGGTATTATAGAGTTGTTTTGAAAATAGAA | ACCCAAACTAACTCCATATTTTTTCC | TTTGGTATTATAGAGTTGTTTTGAAAATAGAA | U-ACCCAAACTAACTCCATATTTTTTCC | TGAAAATAGAAGATAGAGGGA |
| *GDNF* | AGGATTGAGAATTTTTGTTTTTGATT | CCAAACCCTAAATTAAACATTAACTCCA | AGGATTGAGAATTTTTGTTTTTGATT | U-CCAAACCCTAAATTAAACATTAACTCCA | TTTTGTTTTTGATTTGTTG |
| *PRDM5* | TGAGGTTTTGGGGTTAGTTT | CRAATCCRTTCCTACCATTC | TGAGGTTTTGGGGTTAGTTT | U-CRAATCCRTTCCTACCATTC | GTTAATTTYGGGTTAATTAG |
| *MLF1* | GGTGAAGTTATAGAGTAATGTTTAATGGGAAAGTA | CCCCACAAAAACTAACCTCCAAT | GGTGAAGTTATAGAGTAATGTTTAATGGGAAAGTA | U-CCCCACAAAAACTAACCTCCAAT | GTAGTTAGGTGTTAAGTATTTTTTAT |
| *IGF2* | GAGGATTAGGGAGGGAAATATAGT | CCCAAACCCCCAAATTATC | GAGGATTAGGGAGGGAAATATAGT | U-CCCAAACCCCCAAATTATC | AATGGTTATTTAGTTTTTAG |
| *CDH1* | GGAATTGTAAAGTATTTGTGAGTTTG | CAAATACCTACAACAACAACAACAAC | GGAATTGTAAAGTATTTGTGAGTTTG | U-CAAATACCTACAACAACAACAACAAC | GGAAGTTAGTTTAGATTTTA |
| *MYOD1* | AATTAGGGGATAGAGGAGTATTGAAAG | ACAACCCTAAACRACTACACTTAACTC | GAAAGTTAGTTTAGAGGTGA | U-ACAACCCTAAACRACTACACTTAACTC | GAGGTTTGGAAAGGG |
| *SLC16A12* | TAGAGGGAGAGGTGGTTTAGGTGAT | CACCCAAATTAAAATCCCAAACTC | TAGAGGGAGAGGTGGTTTAGGTGAT | U-CACCCAAATTAAAATCCCAAACTC | AAGGGTATTTTTTAAGGAAG |
| *MIR124A1* | AAAGGTGAAAGAAAGGAAGAGG | TCTCCCACTTCCACCCACA | GGGTGGGTAGAAGATGGAATAA | U-CCACCAAAAAAATACTATAATCCC | AATTAGGAAAAAGAAATAAA |
| U =biotin labeled universal primer tag: 5'-biotin-GGGACACCGCTGATCGTTTA | | |  |  |  |

| **Supplementary Table 2** Prevalence of *H. pylori* infection, gastric cancer, inflammatory mucosa and atrophic | | | | |
| --- | --- | --- | --- | --- |
| mucosa in normal and types 1 to 3 gastric mucosal patterns using magnifying NBI endoscopy | | | | |
| Variables | Normal | Type1 | Type2 | Type3 |
| Total number: n | 23 | 15 | 34 | 22 |
| *H. pylori* positives n (%) | 0 (0%) | 13 (86.7%) | 34 (100%) | 5 (23.8%)^$^ |
| Gastric cancer: n (%) | 0 (0%) | 0 (0%) | 3 (8.8%) | 20 (90.9%) |
| Inflammatory mucosa*: n (%) | 0 (0%) | 3 (20%) | 27 (79.4%) | 16 (72.7%) |
| Atrophic mucosa*: n (%) | 0 (0%) | 0 (0%) | 1 (2.9%) | 20 (90.9%) |
| ^$^ For one case, *H. pylori* status was not determined | | |  |  |
| * Inflammatory mucosa was defined as having a score of 2 or higher of acute or chronic inflammation. | | | | |
| Atrophic mucosa was defined as having a score of 2 or higher of atrophy or metaplasia. | | | | |
| These scores were assessed using the updated Sydney system. | | |  |  |

| **Supplementary Table 3** Univariate analysis assessing the factors related to methylation-high cases | | | |
| --- | --- | --- | --- |
| Variables | Odds ratio (95% confident interval) | *P* value |  |
| NBI pattern | 4.04 (2.12-7.7) | <0.0001 |  |
| Age | 1.03 (0.99-1.06) | 0.16 |  |
| Gender (male) | 4.15 (1.29-13.34) | 0.02 |  |
| Gastric cancer occurrence | 3.83 (1.43-10.23) | 0.007 |  |
| *H. pylori* infection | 2.10 (0.85-5.18) | 0.11 |  |
| Inflammatory mucosa | 7.77 (2.77-21.77) | <0.0001 |  |
| Atrophic mucosa | 4.97 (1.77-13.90) | 0.002 |  |
| *Methylation high, mean Z score of methylation >0.15; | |  |  |
| *Mean Z score of methylation more than 0.15 was considered methylation high. | | | |

**Supplementary Table 4.**

**Summary of gene ontology analysis using gradually methylated genes**

|  | | |  |  |  |
| --- | --- | --- | --- | --- | --- |
| Category | Term | Count | % | P-Value | Benjamini |
| INTERPRO | Zinc finger, C2H2-type/integrase, DNA-binding | 12 | 30 | 1.1E-08 | 0.00000073 |
| UP_SEQ_FEATURE | zinc finger region:C2H2-type 9 | 10 | 25 | 3.1E-08 | 0.0000023 |
| UP_SEQ_FEATURE | zinc finger region:C2H2-type 5 | 11 | 27.5 | 0.00000005 | 0.0000023 |
| UP_SEQ_FEATURE | zinc finger region:C2H2-type 16 | 7 | 17.5 | 4.3E-08 | 0.0000024 |
| UP_SEQ_FEATURE | zinc finger region:C2H2-type 12 | 9 | 22.5 | 2.3E-08 | 0.0000026 |
| UP_SEQ_FEATURE | zinc finger region:C2H2-type 8 | 10 | 25 | 7.4E-08 | 0.0000028 |
| UP_SEQ_FEATURE | zinc finger region:C2H2-type 13 | 8 | 20 | 0.0000001 | 0.0000029 |
| UP_SEQ_FEATURE | zinc finger region:C2H2-type 1; degenerate | 7 | 17.5 | 0.00000012 | 0.000003 |
| UP_SEQ_FEATURE | zinc finger region:C2H2-type 4 | 11 | 27.5 | 9.4E-08 | 0.0000031 |
| INTERPRO | Zinc finger, C2H2-like | 12 | 30 | 0.00000014 | 0.0000031 |
| UP_SEQ_FEATURE | zinc finger region:C2H2-type 7 | 10 | 25 | 0.00000014 | 0.0000033 |
| UP_SEQ_FEATURE | zinc finger region:C2H2-type 15 | 7 | 17.5 | 0.00000021 | 0.0000039 |
| INTERPRO | Zinc finger, C2H2-type | 12 | 30 | 0.00000012 | 0.000004 |
| UP_SEQ_FEATURE | zinc finger region:C2H2-type 10 | 9 | 22.5 | 0.0000002 | 0.0000041 |
| UP_SEQ_FEATURE | zinc finger region:C2H2-type 14 | 8 | 20 | 1.8E-08 | 0.0000042 |
| SMART | ZnF_C2H2 | 12 | 30 | 0.00000035 | 0.0000069 |
| UP_SEQ_FEATURE | zinc finger region:C2H2-type 11 | 8 | 20 | 0.0000013 | 0.000022 |
| UP_SEQ_FEATURE | zinc finger region:C2H2-type 3 | 10 | 25 | 0.0000021 | 0.000034 |
| UP_SEQ_FEATURE | domain:KRAB | 8 | 20 | 0.0000029 | 0.000045 |
| UP_SEQ_FEATURE | zinc finger region:C2H2-type 6 | 9 | 22.5 | 0.0000034 | 0.000049 |
| INTERPRO | Krueppel-associated box | 8 | 20 | 0.000004 | 0.000065 |
| SMART | KRAB | 8 | 20 | 0.0000095 | 0.000095 |
| UP_SEQ_FEATURE | zinc finger region:C2H2-type 2 | 9 | 22.5 | 0.000017 | 0.00023 |
| SP_PIR_KEYWORDS | zinc-finger | 13 | 32.5 | 0.000055 | 0.0022 |
| GOTERM_MF_FAT | ion binding | 20 | 50 | 0.00014 | 0.0028 |
| GOTERM_MF_FAT | cation binding | 20 | 50 | 0.00011 | 0.0034 |
| SP_PIR_KEYWORDS | zinc | 14 | 35 | 0.00013 | 0.0034 |
| SP_PIR_KEYWORDS | metal-binding | 17 | 42.5 | 0.00005 | 0.0039 |
| GOTERM_MF_FAT | metal ion binding | 20 | 50 | 0.0001 | 0.0059 |
| GOTERM_MF_FAT | zinc ion binding | 14 | 35 | 0.00045 | 0.0066 |
| GOTERM_MF_FAT | transition metal ion binding | 15 | 37.5 | 0.00078 | 0.0092 |
| SP_PIR_KEYWORDS | dna-binding | 12 | 30 | 0.00055 | 0.011 |
| GOTERM_MF_FAT | DNA binding | 13 | 32.5 | 0.0019 | 0.019 |
| SP_PIR_KEYWORDS | Transcription | 11 | 27.5 | 0.0047 | 0.07 |
| UP_SEQ_FEATURE | zinc finger region:C2H2-type 18 | 3 | 7.5 | 0.0072 | 0.088 |
| SP_PIR_KEYWORDS | Signal-anchor | 5 | 12.5 | 0.0084 | 0.1 |
| UP_SEQ_FEATURE | zinc finger region:C2H2-type 17 | 3 | 7.5 | 0.011 | 0.13 |
| SP_PIR_KEYWORDS | transcription regulation | 10 | 25 | 0.013 | 0.13 |
| PIR_SUPERFAMILY | PIRSF005557:sialyltransferase | 2 | 5 | 0.026 | 0.16 |
| PIR_SUPERFAMILY | PIRSF005557:Sialyl_trans | 2 | 5 | 0.026 | 0.16 |
| UP_SEQ_FEATURE | zinc finger region:C2H2-type 1 | 5 | 12.5 | 0.02 | 0.21 |
| PIR_SUPERFAMILY | PIRSF005559:zinc finger protein ZFP-36 | 3 | 7.5 | 0.02 | 0.23 |
| GOTERM_MF_FAT | sialyltransferase activity | 2 | 5 | 0.042 | 0.31 |
| INTERPRO | Sialyltransferase | 2 | 5 | 0.03 | 0.33 |
| INTERPRO | Glycosyl transferase, family 29 | 2 | 5 | 0.038 | 0.34 |
| KEGG_PATHWAY | Heparan sulfate biosynthesis | 2 | 5 | 0.03 | 0.35 |
| SP_PIR_KEYWORDS | transferase | 7 | 17.5 | 0.048 | 0.38 |
| OMIM_DISEASE | A Genome-Wide Association Study Identifies Protein Quantitative Trait Loci (pQTLs) | 2 | 5 | 0.04 | 0.39 |
| GOTERM_CC_FAT | intrinsic to Golgi membrane | 2 | 5 | 0.049 | 0.43 |
| UP_SEQ_FEATURE | topological domain:Lumenal | 4 | 10 | 0.054 | 0.45 |
| GOTERM_CC_FAT | Golgi apparatus | 4 | 10 | 0.065 | 0.46 |
| GOTERM_CC_FAT | intrinsic to membrane | 11 | 27.5 | 0.03 | 0.49 |
| GOTERM_CC_FAT | integral to Golgi membrane | 2 | 5 | 0.046 | 0.51 |
| UP_SEQ_FEATURE | zinc finger region:C2H2-type 20 | 2 | 5 | 0.067 | 0.51 |
| KEGG_PATHWAY | Glycosphingolipid biosynthesis | 2 | 5 | 0.029 | 0.56 |
| GOTERM_CC_FAT | integral to membrane | 11 | 27.5 | 0.023 | 0.65 |
| GOTERM_BP_FAT | transcription | 11 | 27.5 | 0.0094 | 0.68 |
| GOTERM_BP_FAT | regulation of transcription | 13 | 32.5 | 0.0049 | 0.7 |
| GOTERM_BP_FAT | regulation of RNA metabolic process | 9 | 22.5 | 0.033 | 0.87 |
| GOTERM_BP_FAT | glycoprotein biosynthetic process | 3 | 7.5 | 0.045 | 0.89 |
| GOTERM_BP_FAT | regulation of transcription, DNA-dependent | 9 | 22.5 | 0.029 | 0.91 |
| GOTERM_BP_FAT | glycoprotein metabolic process | 3 | 7.5 | 0.069 | 0.94 |

**Supplementary Table 5.**

Summary of gene ontology analysis using methylated genes only in the type 3 NBI pattern

| Category | Term | Count | % | P-Value | Benjamini |
| --- | --- | --- | --- | --- | --- |
| UP_SEQ_FEATURE | zinc finger region:C2H2-type 10 | 4 | 6.6 | 0.065 | 0.9 |
| UP_SEQ_FEATURE | zinc finger region:C2H2-type 4 | 5 | 8.2 | 0.075 | 0.9 |
| KEGG_PATHWAY | Leukocyte transendothelial migration | 3 | 4.9 | 0.041 | 0.91 |
| UP_SEQ_FEATURE | zinc finger region:C2H2-type 9 | 4 | 6.6 | 0.086 | 0.91 |
| UP_SEQ_FEATURE | domain:KRAB | 4 | 6.6 | 0.064 | 0.93 |
| PIR_SUPERFAMILY | PIRSF005559:zinc finger protein ZFP-36 | 3 | 4.9 | 0.072 | 0.93 |
| UP_SEQ_FEATURE | zinc finger region:C2H2-type 6 | 5 | 8.2 | 0.046 | 0.94 |
| INTERPRO | Intermediate filament, DNA-binding region | 2 | 3.3 | 0.02 | 0.95 |
| UP_SEQ_FEATURE | zinc finger region:C2H2-type 5 | 5 | 8.2 | 0.062 | 0.95 |
| INTERPRO | Krueppel-associated box | 4 | 6.6 | 0.082 | 0.96 |
| UP_SEQ_FEATURE | zinc finger region:C2H2-type 3 | 6 | 9.8 | 0.029 | 0.97 |
| UP_SEQ_FEATURE | zinc finger region:C2H2-type 1; degenerate | 3 | 4.9 | 0.045 | 0.98 |
| GOTERM_BP_FAT | regulation of alpha-beta T cell differentiation | 2 | 3.3 | 0.063 | 0.98 |
| GOTERM_BP_FAT | regulation of leukocyte activation | 3 | 4.9 | 0.068 | 0.98 |
| GOTERM_BP_FAT | positive regulation of transcription from RNA polymerase II promoter | 4 | 6.6 | 0.07 | 0.98 |
| GOTERM_BP_FAT | regulation of cell activation | 3 | 4.9 | 0.075 | 0.98 |
| INTERPRO | Zinc finger, C2H2-like | 6 | 9.8 | 0.078 | 0.98 |
| GOTERM_BP_FAT | regulation of transporter activity | 2 | 3.3 | 0.082 | 0.98 |
| GOTERM_BP_FAT | regulation of alpha-beta T cell activation | 2 | 3.3 | 0.089 | 0.98 |
| GOTERM_BP_FAT | lymphocyte activation | 3 | 4.9 | 0.093 | 0.98 |
| GOTERM_BP_FAT | positive regulation of apoptosis | 4 | 6.6 | 0.099 | 0.98 |
| GOTERM_BP_FAT | regulation of specific transcription from RNA polymerase II promoter | 3 | 4.9 | 0.024 | 0.99 |
| GOTERM_BP_FAT | regulation of gene-specific transcription | 3 | 4.9 | 0.047 | 0.99 |
| GOTERM_BP_FAT | intermediate filament-based process | 2 | 3.3 | 0.055 | 0.99 |
| GOTERM_BP_FAT | regulation of lymphocyte activation | 3 | 4.9 | 0.056 | 0.99 |
| GOTERM_BP_FAT | brown fat cell differentiation | 2 | 3.3 | 0.06 | 0.99 |
| GOTERM_BP_FAT | positive regulation of growth | 3 | 4.9 | 0.018 | 1 |
| GOTERM_BP_FAT | leukocyte activation | 4 | 6.6 | 0.024 | 1 |
| UP_SEQ_FEATURE | zinc finger region:C2H2-type 2 | 6 | 9.8 | 0.026 | 1 |
| GOTERM_BP_FAT | cell activation | 4 | 6.6 | 0.038 | 1 |
| GOTERM_BP_FAT | leukocyte differentiation | 3 | 4.9 | 0.045 | 1 |
| GOTERM_CC_FAT | intrinsic to organelle membrane | 3 | 4.9 | 0.054 | 1 |
| INTERPRO | Zinc finger, C2H2-type | 6 | 9.8 | 0.074 | 1 |
| GOTERM_CC_FAT | chromatin | 3 | 4.9 | 0.094 | 1 |

**Supplementary Table 6.**

**Summary of gene ontology analysis using demethylated genes in the type 3 NBI pattern**

| Category | Term | Count | % | P-Value | Benjamini |
| --- | --- | --- | --- | --- | --- |
| BBID | 70.Signal_peptides_(MHC)_class_I_molecules | 2 | 2.4 | 0.017 | 0.049 |
| GOTERM_CC_FAT | insoluble fraction | 11 | 13.1 | 0.0013 | 0.068 |
| GOTERM_CC_FAT | membrane fraction | 11 | 13.1 | 0.00099 | 0.1 |
| GOTERM_CC_FAT | cell fraction | 11 | 13.1 | 0.0082 | 0.26 |
| KEGG_PATHWAY | Endocytosis | 5 | 6 | 0.012 | 0.43 |
| KEGG_PATHWAY | Circadian rhythm | 2 | 2.4 | 0.062 | 0.79 |
| PIR_SUPERFAMILY | PIRSF001990:class I histocompatibility antigen | 2 | 2.4 | 0.037 | 0.81 |
| GOTERM_MF_FAT | stearoyl-CoA 9-desaturase activity | 2 | 2.4 | 0.013 | 0.92 |
| GOTERM_MF_FAT | oxidoreductase activity, acting on paired donors, with oxidation of a pair of donors resulting in the reduction of molecular oxygen to two molecules of water | 2 | 2.4 | 0.027 | 0.92 |
| SMART | C345C | 2 | 2.4 | 0.083 | 0.94 |
| OMIM_DISEASE | Loci influencing lipid levels and coronary heart disease risk in 16 European population cohorts | 2 | 2.4 | 0.084 | 0.94 |
| GOTERM_MF_FAT | insulin receptor substrate binding | 2 | 2.4 | 0.048 | 0.96 |
| GOTERM_MF_FAT | MHC class I receptor activity | 2 | 2.4 | 0.073 | 0.97 |
| INTERPRO | EGF | 3 | 3.6 | 0.1 | 0.98 |
| INTERPRO | Netrin module, non-TIMP type | 2 | 2.4 | 0.069 | 0.99 |
| SMART | LamNT | 2 | 2.4 | 0.078 | 0.99 |
| INTERPRO | Fps/Fes/Fer/CIP4 homology | 2 | 2.4 | 0.085 | 0.99 |
| INTERPRO | Netrin domain | 2 | 2.4 | 0.092 | 0.99 |
| INTERPRO | Fatty acid desaturase, type 1 | 2 | 2.4 | 0.033 | 1 |
| UP_SEQ_FEATURE | short sequence motif:Histidine box-3 | 2 | 2.4 | 0.035 | 1 |
| UP_SEQ_FEATURE | short sequence motif:Histidine box-2 | 2 | 2.4 | 0.035 | 1 |
| UP_SEQ_FEATURE | short sequence motif:Histidine box-1 | 2 | 2.4 | 0.035 | 1 |
| UP_SEQ_FEATURE | region of interest:Alpha-3 | 2 | 2.4 | 0.035 | 1 |
| UP_SEQ_FEATURE | compositionally biased region:Pro-rich | 9 | 10.7 | 0.037 | 1 |
| SP_PIR_KEYWORDS | mhc i | 2 | 2.4 | 0.039 | 1 |
| GOTERM_BP_FAT | neuron development | 5 | 6 | 0.049 | 1 |
| GOTERM_BP_FAT | anti-apoptosis | 4 | 4.8 | 0.051 | 1 |
| GOTERM_BP_FAT | regulation of protein complex assembly | 3 | 3.6 | 0.052 | 1 |
| UP_SEQ_FEATURE | domain:Laminin N-terminal | 2 | 2.4 | 0.062 | 1 |
| SP_PIR_KEYWORDS | heterodimer | 3 | 3.6 | 0.064 | 1 |
| INTERPRO | Laminin, N-terminal | 2 | 2.4 | 0.065 | 1 |
| GOTERM_BP_FAT | antigen processing and presentation of peptide antigen via MHC class I | 2 | 2.4 | 0.067 | 1 |
| UP_SEQ_FEATURE | region of interest:Alpha-1 | 2 | 2.4 | 0.077 | 1 |
| UP_SEQ_FEATURE | region of interest:Alpha-2 | 2 | 2.4 | 0.077 | 1 |
| UP_SEQ_FEATURE | domain:FCH | 2 | 2.4 | 0.077 | 1 |
| UP_SEQ_FEATURE | domain:Laminin EGF-like 3 | 2 | 2.4 | 0.084 | 1 |
| UP_SEQ_FEATURE | domain:NTR | 2 | 2.4 | 0.084 | 1 |
| GOTERM_BP_FAT | neuron projection development | 4 | 4.8 | 0.086 | 1 |
| UP_SEQ_FEATURE | domain:Laminin EGF-like 1 | 2 | 2.4 | 0.098 | 1 |

**
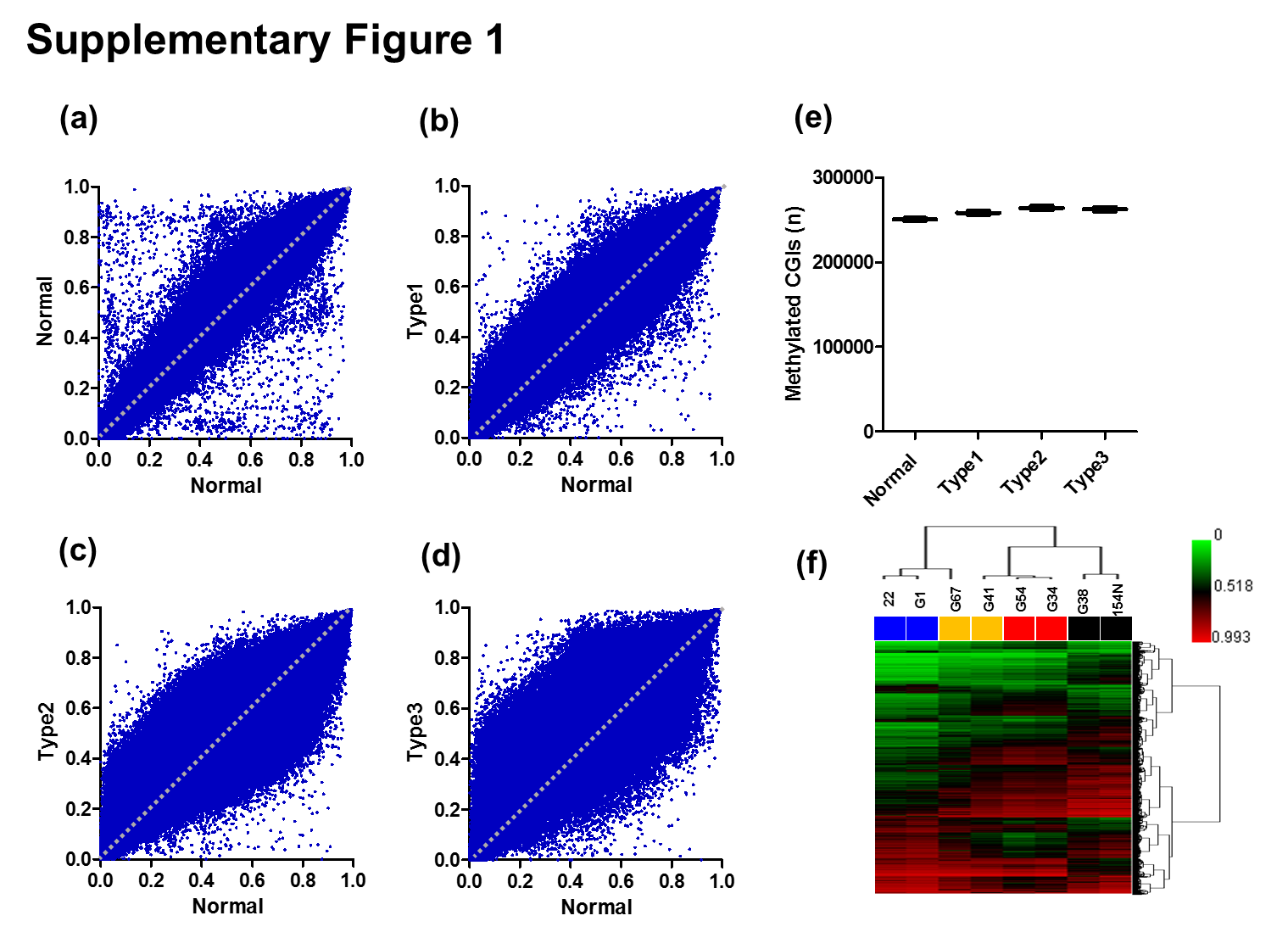
**

**
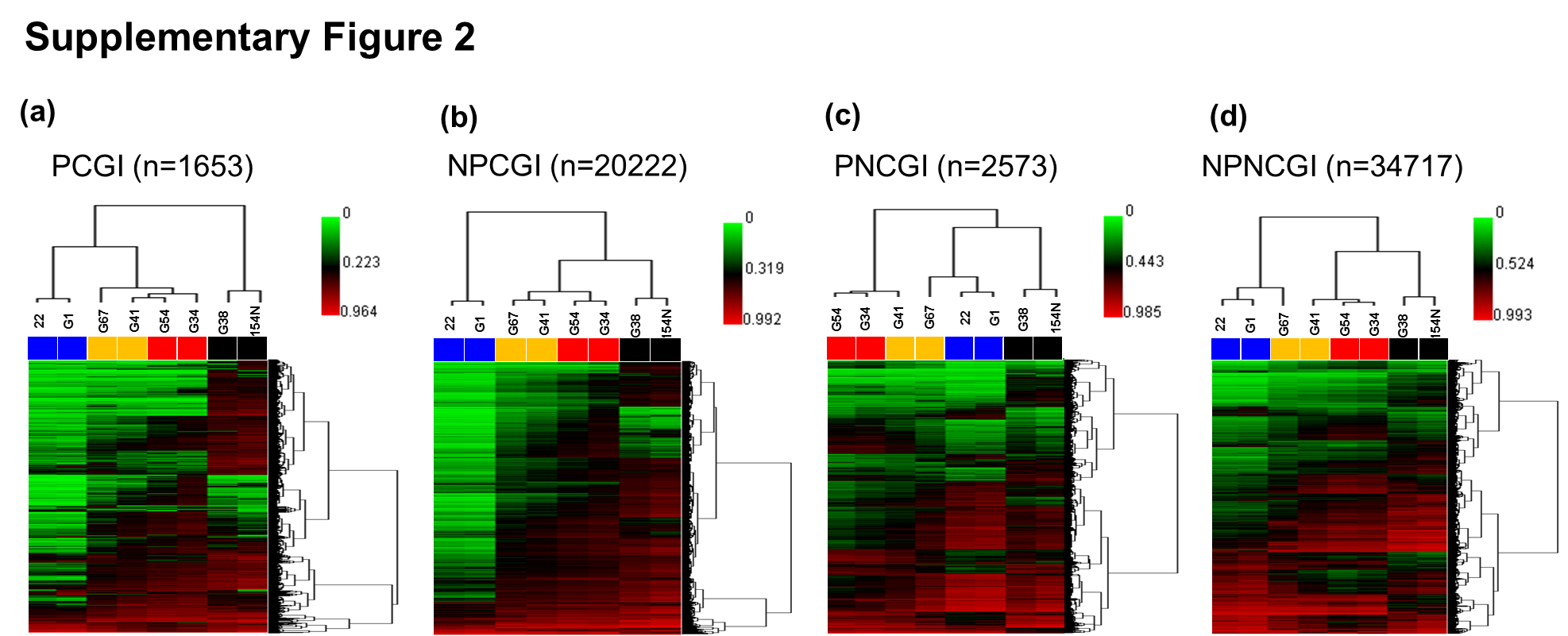
**

**
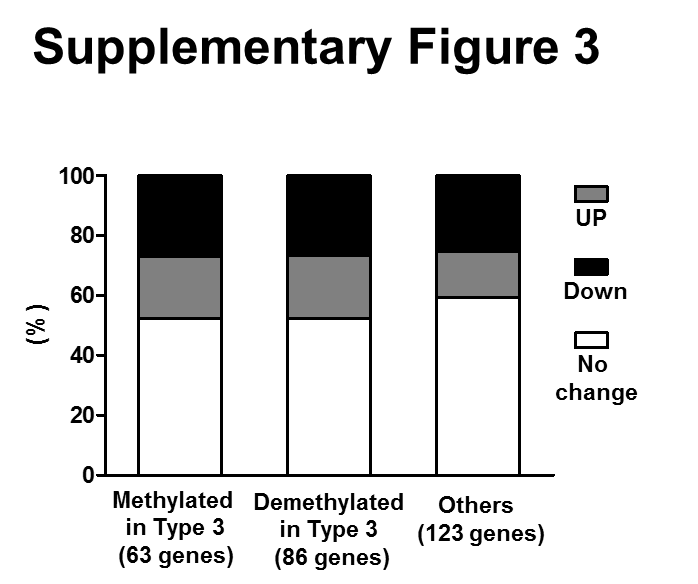
**

**Supplementary Figure 1** Genome-wide methylation analysis in outside CpG islands

(NCGIs).

a-d: The development of mucosal patterns from normal to types 1, 2, and 3, and methylation accumulation in NCGIs.

e: Mean number of methylated sites in the NCGIs from normal to types 1, 2, and 3.

f: Unsupervised hierarchical clustering analysis of 10% of the most variant probes. Blue, yellow, red and black boxes represent normal and type 1, 2, and 3 samples, respectively. Sample ID numbers are listed above the boxes.

**Supplementary Figure 2**

Unsupervised hierarchical clustering analysis using 10% of the most variant probes.

All sites were divided into promoter CGI (PCGI: a), outside promoter CGI (NPCGI: b), promoter NCGI (PNCGI: c) and outside promoter NCGI (NPNCGI: d).

**Supplementary Figure 3**

Gene expression fold change between normal and type 3 among genes that were strikingly hypermethylated (63 genes) and hypomethylated (86 genes) only in type 3 compared with the remaining genes (123 genes). Statistical analysis was performed using chi square test.
